# Over the Hill at 24: Persistent Age-Related Cognitive-Motor Decline in Reaction Times in an Ecologically Valid Video Game Task Begins in Early Adulthood

**DOI:** 10.1371/journal.pone.0094215

**Published:** 2014-04-09

**Authors:** Joseph J. Thompson, Mark R. Blair, Andrew J. Henrey

**Affiliations:** 1 Department of Psychology, Simon Fraser University, Burnaby, British Columbia, Canada; 2 Department of Statistics and Actuarial Science, Simon Fraser University, Burnaby, British Columbia, Canada; University of California, San Francisco, United States of America

## Abstract

Typically studies of the effects of aging on cognitive-motor performance emphasize changes in elderly populations. Although some research is directly concerned with when age-related decline actually begins, studies are often based on relatively simple reaction time tasks, making it impossible to gauge the impact of experience in compensating for this decline in a real world task. The present study investigates age-related changes in cognitive motor performance through adolescence and adulthood in a complex real world task, the real-time strategy video game StarCraft 2. In this paper we analyze the influence of age on performance using a dataset of 3,305 players, aged 16-44, collected by Thompson, Blair, Chen & Henrey [1]. Using a piecewise regression analysis, we find that age-related slowing of within-game, self-initiated response times begins at 24 years of age. We find no evidence for the common belief expertise should attenuate domain-specific cognitive decline. Domain-specific response time declines appear to persist regardless of skill level. A second analysis of dual-task performance finds no evidence of a corresponding age-related decline. Finally, an exploratory analyses of other age-related differences suggests that older participants may have been compensating for a loss in response speed through the use of game mechanics that reduce cognitive load.

## Introduction

Among the general public, people tend to think of middle age as being roughly 45 years of age, after which there are obvious age-related declines in cognitive-motor functioning. Once “over the hill”, experience and wisdom, the consolation prizes of age, are hoped to be sufficient to either attenuate this decline or at least compensate for it indirectly. Aging research has shown that this general conception is incorrect. There is much evidence that memory and speed on a variety of cognitive tasks may peak much earlier [2], [3], [4], [5]. However, the pervasive intuition may still have merit if declines are restricted to laboratory tasks and are not noticeable in, or relevant to, real world performance. A complete understanding of the “over-the-hill” intuition would therefore seem to require a look for age-related declines in direct measures of real world performance.

The typical challenges in studying real world behavior are exacerbated in the study of aging, however, as almost all natural task environments are rife with structural regularities that aging individuals could use to compensate for cognitive decline. In many cases, age will presumably allow for skill development that is more pronounced than any age-related decline associated with the skill. For example, academic psychologists seem to be most productive at 40 years of age [6], suggesting that any earlier age-related decline is trumped by skill development. Unfortunately, the simple lab based tasks used in most studies remove any possibility for compensatory strategies, and thus obfuscate the cognitive system's natural compensatory capacities. Assessing whether a deficit has any real world relevance would seem to require large samples with a variety of measures so that possible compensatory mechanisms can be identified.

There are several ways in which experience can compensate for age-related deficits. First, older participants can develop different approaches to relevant tasks, such that they can attenuate specific declines in performance directly. For example, though older typists show declines in finger tapping tasks there is no evidence for a decline in typing speed with age [7]. Research suggests that older expert typists accomplish this by looking farther ahead, and thus allowing additional time for motor preparation [7], [8]. Participants with college degrees seemed to have reduced declines on certain reaction time task over the phone [9]. In other cases, the original age-related decline can be reduced but not necessarily eliminated by expertise, as in flight simulator control precision [10] or in piano-related performance [11]. Experience can also *indirectly* compensate for age-related deficits by improving other areas of performance, so that overall performance does not suffer, even though the specific deficits remain. In chess tasks involving check threat detection experts seem to suffer as much as novices from age-related decline [12]. However, older chess experts can obviously retain high levels of general performance despite specific unattenuated declines.

There are few data that can offer fair assessment of the “over-the-hill” intuition. Most aging studies are aimed primarily at charting the overall trajectory of cognitive-motor declines across the entire adult lifespan, with a particular interest in the elderly. While this is, of course, a sensible research approach, it is ill-suited to discerning the onset of cognitive-motor declines and identifying potential compensatory mechanisms in young adulthood. Declines, if they exist in early adulthood at all, are likely to be small, and aging studies seldom have a sufficiently large sample of participants concentrated on the ages of interest, roughly 16–45 years. Also, analyses in these studies are typically simple linear regressions that, by definition, assume linear change starting at the youngest ages in the sample. While this approach can establish overall change across age, it is not appropriate for pinpointing the onset of declines.

**Figure 1:**
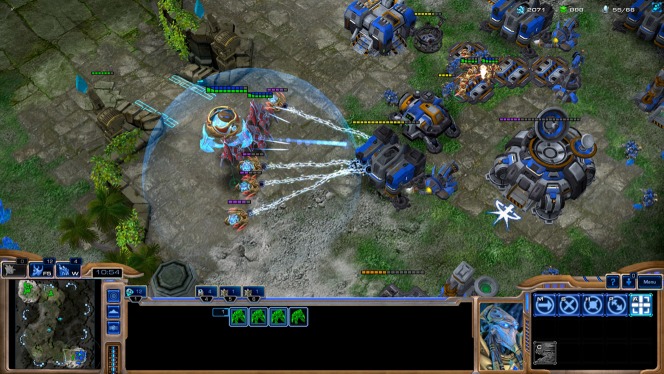
A screenshot from the game StarCraft 2.

The present study investigates the onset of age-related declines in cognitive motor speed and dual-task performance and explores how domain experience may compensate for this decline. We overcome the limitations of prior studies by using data collected from players of the real-time strategy video game StarCraft 2 (Figure 1). Like chess, the game's objective is to defeat the opponent's army. Doing so requires analogous considerations regarding the movement of one's army. However, StarCraft 2 players are also responsible for managing their civilization's game economy and military production, and for choosing whether to emphasize military or economic growth. Furthermore, all aspects of StarCraft 2 occur in real-time, allowing players to give commands as fast as they can use the game interface. This means that, while each individual game action probably does not involve the sort of careful decision making as moves in chess, players must make a myriad of dynamic adjustments in order to ensure the implementation of a larger plan. Finally, attentional allocation plays a special role in StarCraft 2, as players can only view a small portion of the game map in detail at any given time. This view-screen is important not only for seeing detailed information regarding the game state (gross information is given in a small ‘mini-map’ in the corner of the player's game interface) but also for manipulating units within the game. StarCraft 2 is therefore a real world task in the same sense that chess, or basketball are real world tasks and a Rapid Serial Visual Presentation task is not. This is not to say that StarCraft 2, at least for most people, is an everyday task in the sense that getting groceries or paying bills is. We only mean that StarCraft 2 exists outside of the laboratory, and elicits the voluntary participation of thousands of people on a daily basis.

This game brings several important advantages to the study of aging. First, the data are derived from a rich, demanding and ecologically valid domain in which players from adolescence to middle age voluntarily participate for many hours per week. This allows us to preserve the availability, and utility of compensatory mechanisms, and consequently allow us to study the impact of declines on ecologically valid measures of complex task performance. This is not to assume that age-related change in StarCraft 2 performance is comparable to change in other real world tasks (indeed, we would be uncomfortable with making such assumptions about any domain, even laboratory-based tasks). The relevance of some aspects of StarCraft 2 to other complex tasks is certainly plausible (especially in technology mediated tasks such as laparoscopic surgery or emergency management, where the latter involves allocation of limited resources to particular locations using software that is not that different from the StarCraft 2 interface), but the extent of the similarity is ultimately an empirical question. Furthermore, we take this to be an empirical question for which the current methods are well suited. Second, because players can have hundreds of hours of experience playing the game, we can assess the degree to which declines, or compensation for declines, relates to experience. Third, StarCraft 2 allows players to save game records for later review. These game records include the data necessary for StarCraft 2 to replay the entire game in detail. The “replay” file is automatically generated after a game is complete, and thus allows noninvasive and direct measures of natural performance rather than laboratory-based analogs of natural performance. In this sense, the study of StarCraft 2 has much in common with the Space Fortress project, a game developed by researchers to study cognition in a more complex task environment [13], [14]. Unlike the Space Fortress game, however, StarCraft 2 allows for a telemetric data collection procedure that supports samples that are far larger, and more geographically diverse than is typically possible. In summary, by using data from StarCraft 2 we are able to collect a large sample of task relevant data from a rich, complex, and real world cognitive motor task with participants of varying experience to produce a clearer estimate of the onset of cognitive decline, to estimate it's domain impact, and to outline potential compensatory strategies used by aging players.

StarCraft 2 is of interest to psychologists for the same reasons that Go, chess, and bridge [15], [16], [17] are interesting. One difference is that StarCraft 2 progresses in real-time, conferring a large advantage to players who can act and make decisions quickly. The role of attention in StarCraft 2 is also slightly different, as players are unable to observe the entire game state at once. Not only must players act under uncertainty of their opponent's movements until one of their units (analogs of chess pieces) are within a close proximity, they can only observe one portion of the game-map in detail at any time (Figure 1). This means that StarCraft 2 players must choose where to allocate their view-screen, which is important both for giving commands to one's units, and also for assessing the current game state. This has allowed researchers to define a self-initiated response time measure (Looking-Doing Latency) in terms of the latency to an action after a new “fixation” of the view-screen [1]. Players typically have about 300 looking-doing cycles per game, and in the present work we consider each player's mean looking-doing latency. The game also imposes dual-task constraints, which are thought to become more problematic with aging [4], [5], as players must extract resources to build units, forcing players to constantly shift between economic and military tasks at regular intervals. This shifting may or may not relate well to laboratory experiments on dual-task performance, but plausibly relate to the real world management of competing interests in tasks such as emergency response. See Lewis, Trinh & Kirsh [18] and Thompson, Blair, Chen & Henrey [1] for a more exhaustive defense of the game's relevance to psychology.

The analysis in the present research addresses a hierarchy of theoretical questions regarding aging in 16–44 year-olds. To what extent does looking-doing latency and dual-task performance in complex tasks show age-related declines and when do these declines occur? If there are age-related declines in performance, might they be ameliorated by expertise as they appear to be in older typists [7], [8]? If there are age-related declines that cannot be directly ameliorated, can players compensate in overall performance through improvement in other areas important to performance, as older chess experts presumably need to compensate for slower threat detection [12].

## Method

### Ethics statement

The current study is based entirely on data acquired by Thompson, Blair, Chen & Henrey (in press). Both studies were reviewed and approved by the Office of Research Ethics at Simon Fraser University (Study Number: 2011s0302). Participants provided informed consent (via a check box) in an online survey.

### Game variables relevant to aging: primary analyses

Previous work found that, depending on the method used to move one's view-screen, the coordinates associated with the change can vary substantially for uninteresting reasons to do with how the game records these locations. To clean up these data we aggregated view-screen movements into view-screen fixations using a Goldberg & Salavucci [19] algorithm, such that screen-fixations are at least 20 game timestamps (roughly 230 milliseconds) in duration, much as one would compile raw eye-tracking data into fixations to specific locations for specific durations. We predicted that looking-doing latency, an excellent predictor of StarCraft 2 expertise [1], would increase with age. The variable is analogous to reaction time [9], in that players are presented with new stimuli as they make new fixations, but differs in that players initiate such changes themselves.

We also included the number of workers trained, a variable hypothesized to measure dual-task performance within StarCraft 2 [1]. Players must produce workers periodically for a healthy economy in StarCraft 2, and these workers typically have no immediate or direct relevance to a player's military goals. Importantly, there are constraints on how many workers can be produced and when (only certain structures can produce workers and most of these can only produce one at a time) and, consequently, failure to remember to produce workers at a regular interval throughout the early and middle of StarCraft 2 games results in a significant loss of potential income. Nevertheless, it is possible, though it seems very unlikely, that some players could focus solely on worker production for half the game and then switch entirely to military production. We hypothesized worker production would, like other forms of dual-task performance [4], [5], show age-related decline.

### Game variables relevant to aging: exploratory analysis of compensation

We considered a number of variables in our exploratory analysis of possible compensatory mechanisms. Importantly, these variables were selected on the basis of a previous analysis of the same dataset that showed these variables to be predictive of league [1]. This has important consequences for the interpretation of our compensation analysis and is discussed below.

First we considered reported hours of experience per week and total reported hours of StarCraft 2 experience. We hypothesized that older experts might be able to compensate for age-related decline with sheer experience.

Secondly, we recorded a set of variables pertaining to actual game performance (for full definitions of all these variables, see Materials S1). Assignment of units to hotkeys allows a player to reselect that set of units quickly, and so Thompson, Blair, Chen & Henrey [1] hypothesized that effective use of hotkeys could allow players to access and organize units and structures relevant to often disparate game tasks (such as combat units, upgrade structures, production units, and production structures) more easily. The frequency of unique hotkeys used, the frequency of hotkey assignments made, and the frequency of using hotkeys to select units are all included in the analysis.

Complex units are ones that require more delicate targeting instructions, so we hypothesized that players could reduce cognitive load by avoiding them. Complex abilities, like complex units, are abilities that, because of the targeting instructions required for their use, might place additional demands on the player.

Players can choose to command their units within their view-screen or to command them using the ‘mini-map,’ which allows them to give certain gross movement commands (called right clicks offscreen, and attacks offscreen) without moving their view-screen. We suspected that players might be able to compensate for increased demands by making more use of this aspect of the games interface.

### Overview of data collection

The present study uses an extensive dataset of game replay files, and survey questions first reported in Thompson, Blair, Chen & Henrey [1]. While full details of the data collection, including sample characteristics, are available in that paper, we shall briefly summarize the data collection here. We recruited participants using a variety of social media websites where StarCraft 2 players are known to frequent. Participants filled out a short survey and provided exactly one replay file. We parsed these game records with free SC2Gears software, providing us with a text file containing every meaningful game action produced by the participant during the game. Data were uploaded to a MYSQL server and MATLAB scripts were then used to extract variable relevant to performance.

Participants provided their Battle.net ID, which allowed us to extract league information that reflects their level of expertise (Bronze; Silver; Gold; Platinum; Diamond; Master; Grandmaster). The game's manufacturer, Blizzard, uses this information to match players against other players of similar skill. The algorithm underlying league placement is complicated but a major determinant is a given player's hidden “match-making rating”, an analog of ELO in chess (Blizzard does not make this rating public), and this makes it a desirable measure of skill for the present study.

**Figure 2:**
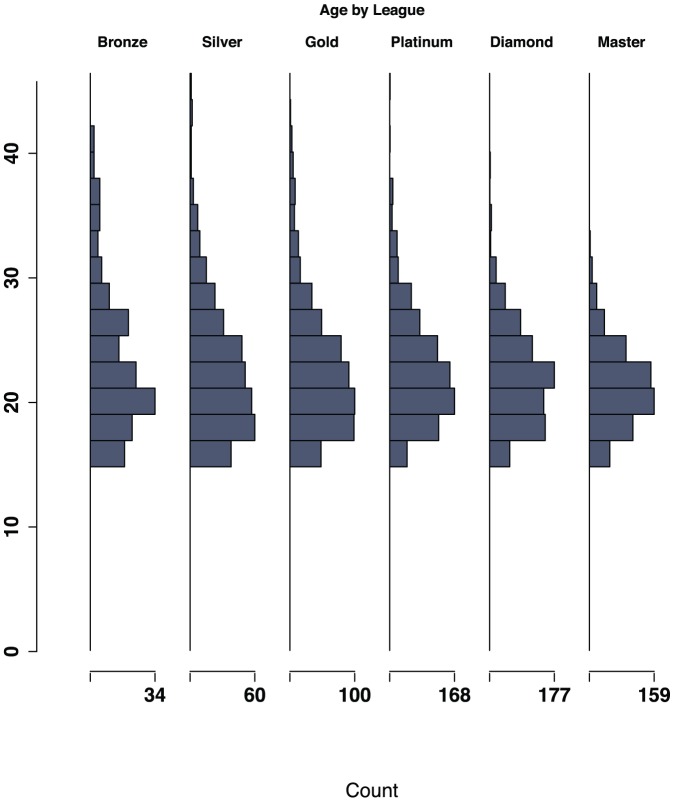
Histogram of Age by league. Leagues, from left to right, are Bronze (n = 167; M = 22.72, SD = 5.22), Silver (n = 347; M = 22.21, SD = 5.17), Gold (n = 553; M = 22.05, SD = 4.9), Platinum (n = 811; M = 21.98, SD = 4.14), Diamond (n = 806; M = 21.36, SD = 3.66), and Masters (n = 621; M = 20.7, SD = 3.02).

**Figure 3:**
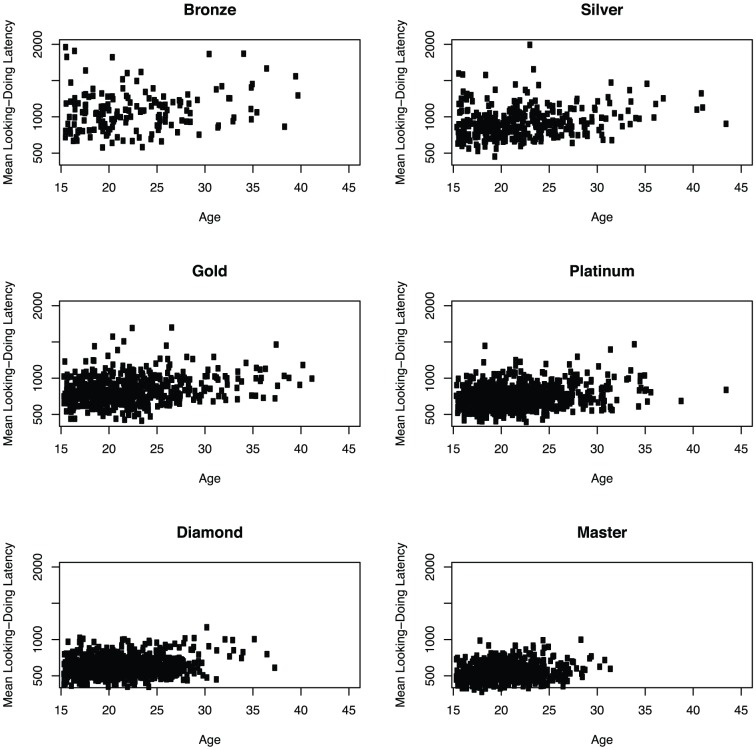
Scatter plots of age and looking-doing latency by league.

The distribution of ages (Mean = 21.7; SD = 4.2) in our sample is reported in Figure 2. Most participants described their country of origin as the United States (1425), Canada (480), Germany (246), or the United Kingdom (187). Finally, the sample includes 3276 males and 29 females, so no generalizations here will be extended to the latter population. Scatter plots of raw age and looking-doing latency are seen in Figure 3.

### Game stability

It is important to mention that StarCraft 2 games can be played in teams, against computers of various difficulties, or with “custom maps” that can deviate from typical multiplayer games in a myriad of ways. In order to guarantee consistency between game starting conditions, we only considered games between two human players where the opponent was selected using Blizzard's match-making system. Players in higher leagues can therefore be expected to generally have higher skilled opponents. The initial conditions of our games can vary in terms of each player's starting location on standard game maps, which are designed to be balanced to all starting positions (e.g., they are symmetrical). There is no reason to think that any slight advantages that might remain could influence the performance measures used in this study.

## Results

### Looking-doing analysis

We attempted to answer three research questions.

1. Is there age-related slowing of Looking-Doing Latency?
2. Can expertise directly ameliorate this decline?
3. When does this decline begin?

We used linear regression to answer these research questions. We begin with a linear model (model 1) of age and skill regressed onto the logarithm of Looking-Doing Latency. We found that the LDL itself as a response is heteroskedastic (we assessed by our residual vs fitted value plot), so we used a log-transformation. This induces a slight non-linearity in the modeled relationship between age and Looking-Doing Latency. While this transformation allowed us a straightforward statistical analysis of the present research questions, it does not permit a straightforward test of whether the relationship between age and LDL is non-linear. Interested researchers will have to employ more appropriate methods for dealing with that research question. We included the interaction of age and skill to test whether skill could attenuate age-related-decline. Age is related to increased Looking-Doing Latency (p<0.01), but the interaction term is not significant. Thus, it appears that there is age-related slowing of looking-doing responses, but that this decline is not ameliorated by level of expertise. See the Materials S1 for details regarding all models described in the present work.

**Figure 4:**
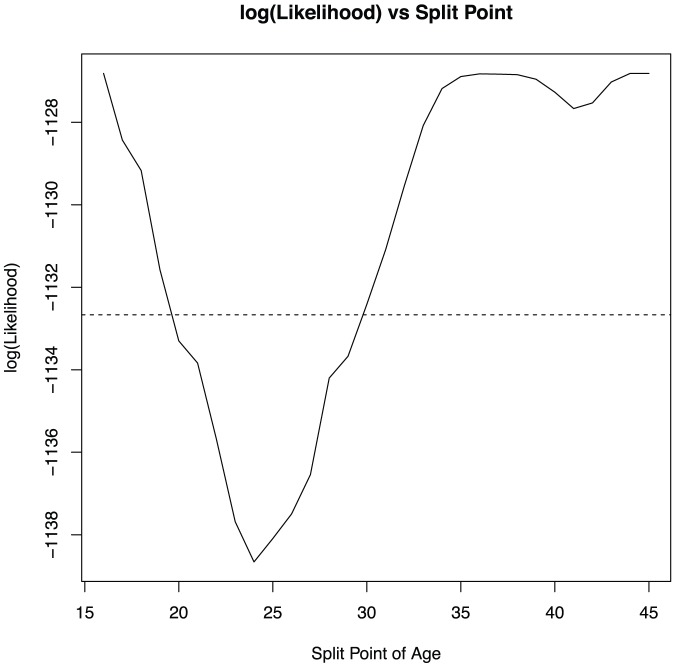
Split-point, K, against log likelihood. Values below the dotted line are part of the confidence region.

To answer the third research question, we consider a piecewise linear model (model 2) where the effect of age on reaction time changes at a certain point: for the first K years, the effect of age on log(looking-doing latency) has slope β1, and afterwards, has a different slope β2. This new model adds two extra parameters (K and β2). We fit the new model using maximum likelihood and evaluate the quality of the fit using a likelihood ratio test against our original model. The likelihood ratio test is justified as the two models are nested – we could set, for example, K = 0 and β1 = β2 to attain the original model. The test statistic is 12.7, and the χ^2^_(0.95,2)_ critical value is 5.99. The piecewise linear model provides significantly better fit than a single slope across all ages (model 1). Figure 4 shows the log likelihood values for the models with each of the possible values of K. Models with K in the twenties have superior fit. The likelihood ratio confidence interval for K is [20,29], and the most likely value of K is 24 (adjusted R^2^ = 0.47).

**Figure 5:**
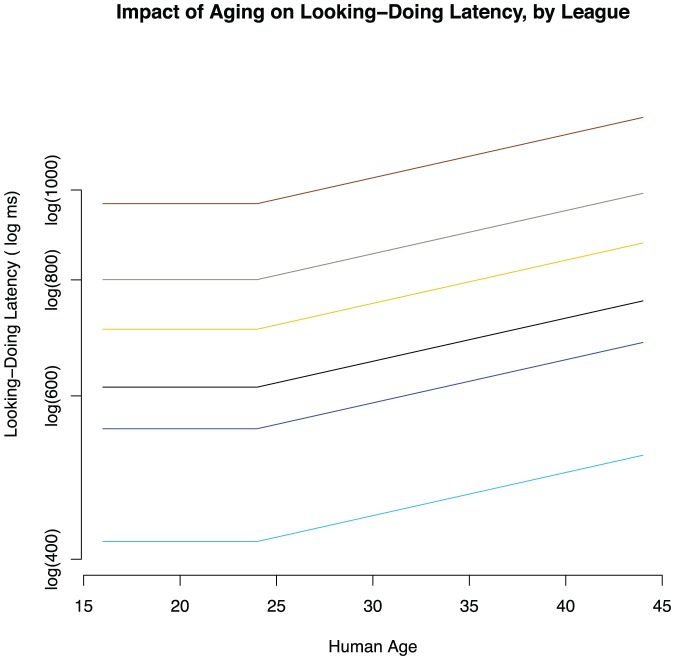
Impact of aging on PAC Latency, and respective intercepts by League as described by the best fitting piecewise linear model. Leagues, from top to bottom, are Bronze, Silver, Gold, Platinum, Diamond, Masters. No evidence for an interaction between league and age was found, and so depicted slopes do not differ by league.

Tests on coefficients in our best model, where K = 24, found intercepts to vary with league (*p*<0.05). However, there was no evidence of a general effect of age (β*_(age)_* not significantly different from zero; p>0.05). Instead, coefficients corresponding to *years of age over 24* were significantly non-zero (β*_(years-over-24)_* is non-zero; *p*<0.05; see Materials S1 for the obtained equations). There was no evidence that the effect of age varied by league (β*_(years-over-24 X League)_* not significantly different from 0; p>0.05). We conclude that age-related decline begins around 24, and probably not outside of the twenties. Figure 5 describes the statistically significant findings.

One possible concern is that our finding of age-related decline in StarCraft 2 could be due to a speed accuracy trade-off: older players become slower in virtue of focusing on accurate movements or strategic planning. It is straightforward to imagine this kind of trade-off in a strategy game like chess, where one could improve one's decisions by spending more time exploring possible moves. In StarCraft 2, it's not clear that speed-accuracy trade-offs of the kind typically discussed even exist. The vast majority of player actions can only be inaccurate in the sense that a player has performed an unintended keystroke or mouse movement. While moving a pawn when one should have moved a rook is often a serious mistake in chess, it is typically not in StarCraft 2, in part because actions can simply be reversed easily, and in part because most actions are made within the player's view-screen (which occupies less than 5% of typical competitive StarCraft 2 maps) and so one often can only err so dramatically, and enemy armies are typically too far apart to capitalize on any such mistakes before they can be corrected. As a result, most of the individual actions in StarCraft 2 are of little strategic significance in and of themselves.

The existence of a speed-accuracy trade-off also seems at odds with our results. StarCraft 2 strategy is much more about implementing plans to build an army of a particular composition, or to be ready to attack at a particular time. Strategy in StarCraft 2 is therefore more like a speed cooking contest where recipes can be planned in advance. When weaker players leave one another unimpeded, StarCraft 2 often becomes a game of who can produce the largest army and best economy first. A much more complex form of strategy exists for better players that are able to impede others while continuing to develop their army. It seems extremely unlikely that the presence of and frequency of this strategizing is constant across StarCraft 2 leagues. Similarly, it seems implausible that the usefulness of precision keyboard and mouse movements is constant across leagues, as higher leagues seem more likely to use units and abilities that require delicate targeting instructions [1]. If our results were due to a speed-accuracy or a speed-strategizing trade-off, then we would expect an interaction between league, age, and looking-doing latency, which we do not find.

Finally, to consider the possible influence of intra-individual variability on our results we created two models that regress the standard deviation of intra-individual looking-doing latencies on league and age respectively. We found an effect of league (p<0.0001), but found no evidence of a linear relationship between age and looking-doing latency standard deviations.

### Workers trained analysis

Our second analysis was the same as analysis 1, except that Workers Trained was used as the dependent variable. There was no evidence that older adults had especial problems with the dual-task demands implied by worker production (*p = 0.97*), so we did not perform a piecewise analysis to answer question 3. There is evidence to expect declines in dual-task performance [4], [5], so it may be that the frequency of training workers is unrelated to the kinds of tasks used in dual-task studies. It is also possible, however, that worker production has already been mastered by the majority of our sample. The typical participant reported a total of 545 hours playing StarCraft 2 (based on a one-tailed 95% trimmed mean), which is about 50 times that of the typical automaticity study [20].

### Exploratory compensation analysis

The slowing of looking-doing latencies imposes a threat to player performance, as looking-doing latency is related to expertise (*p*<0.001). Furthermore, older StarCraft 2 experts do not seem to have any effective strategies for directly ameliorating this cognitive-motor decline as typists do. However, as StarCraft 2 is a more complex task environment than is present in typical laboratory studies, we hypothesized that older StarCraft 2 players could compensate for this decline by improving performance in other aspects of the game.

We constructed 10 linear regression models (models 3–12) with compensatory variables as dependent variables and age and league as predictors. Because these compensatory variables were discovered [1] to be related to skill using the same data set that we are using now, the absolute p-values produced by any additional analysis may be somewhat inflated. However, because our data set is large and the number of parameters in question is small, the additional inflation is unlikely to be too strong. The main cause of concern is that adding these parameters to the model via variable selection gives us a biased estimate of the mean squared error. Hastie, Tibshirani, & Friedman [21] show that the error is optimistic by a factor of at most 2*P/N %. Our previous model with 16 parameters has an n of 3305. As such, we don't think that variable selection from our previous analysis should have much bearing (at most about 1%) on p-values. Nevertheless, we view our analysis as an exploratory one with the goal of indicating plausible compensatory candidates. We therefore do not control for familywise type I error and report potentially biased p* values. Where p* values were greater than 0.05 we do not report the sign.

On two measures, older players showed signs of being more advanced than they actually are. Both Unique Hotkeys per game timestamp (more with age; p*<0.001), and Offscreen attacks per game timestamp (more with age; p*<0.001) were strong candidates as compensators. Older players in our sample exhibited more impressive hotkey performance, even when skill was controlled for, suggesting that our participants may be indirectly compensating for declines by offloading demands to the game interface. An increase in attacks to areas outside of the view-screen might reflect heightened awareness of global game information via attention to the ‘mini-map’. Generally then, older players seem better at using available interface features (customizable keys and the ‘mini-map’) than younger players.

By other measures, older players show weaker performance than younger players (again, controlling for league). First, despite using a larger variety of customizable hotkeys, older players assign units to hotkeys less often - Hotkey Assigns per game timestamp (fewer with age; p* = 0.005). This is possible because during the gameplay new units are constantly created, and thus need to be to added to existing hotkey groups. Older players seem worse at this kind of hotkey maintenance. Similarly, older players seem to actually use their hotkeys to select units less often than younger players - Hotkey Selects per game timestamp (fewer with age; p*<0.001). Consequently, the more unique hotkeys used by older players noted above does not seem to convey a benefit by speeding selection of units generally. Assigning a greater variety of hotkeys may be beneficial to older players as a kind of memory aid, allowing players to remember to upgrade units (by making a special hotkey for upgrades), or do other important but low frequency game actions.

Another difference in play that is related to age is the complexity of both the units and abilities used during the game - Complex Units made per game timestamp (fewer with age; p*<0.001), Complex Abilities used per game timestamp (fewer with age; p* = 0.002). Older players seem to prefer simpler abilities and units compared to their younger counterparts. While this could be interpreted as poorer performance, because complex unit/ability use generally increases with experience, it might also be that older players are choosing easier to execute strategies as a way to divert cognitive resources to other, perhaps more important, tasks.

Finally, there is no evidence that age is related to Offscreen right clicks per game timestamp (p* = 0.683), or that age is related to the Total Hours of experience reported (p* = 0.430). Older players do report playing fewer hours per week, however (fewer with age; p*<0.001).

## Discussion

In an article entitled “When does cognitive aging begin?” Salthouse [2], summarized the available aging evidence and concluded that the correct answer is that general cognitive decline begins in the 20 s and 30 s. The present study, employing performance measures from thousands of video game players, provides a more precise estimate: cognitive decline begins around 24.

One argument in favor of ignoring aging in young adulthood is that declines at that age are small and have no real world impact. However, there can be no contention that increases in looking-doing latency are of significance to complex human performance outside of the laboratory. Analysis 1 shows that looking-doing latency is related to skill, and an independent analysis in Thompson, Blair, Chen & Henrey [1] showed that looking-doing latency (which they termed first action latency) is, of the 15 variables they investigated, one of the single best predictors of a player's league. The effect of age is substantial. For example, a typical Bronze player at the age of 39, equal in all other respects to a 24 year-old adversary, can be expected to be around 150 milliseconds slower in their typical looking-doing latencies, costing about 30 seconds over a typical 15 minute Bronze game containing 200 looking-doing cycles. This is a long time in a game of speed such as StarCraft 2. More generally, the effect of age is comparable even to large changes in skill. After 24, the expected slowing due to an additional 15 years of age amounts to about 15% of the speed enjoyed by professional players over bronze ones. That is, the effect of age, even in what most consider young adulthood, can be expected to offset a sizeable proportion of what has taken older players hundreds or even thousands of hours to achieve.

Our response time measure, looking-doing latency, is an ecologically valid *analog* of reaction time and, we would argue, more useful than simpler reaction time measures. Responses in the real world are embedded in complex and dynamic situations with a myriad of informative regularities. Even a situation as simple pressing the accelerator when the traffic light turns green has a rich set of regularities: the typical duration of that specific light, the density of cross traffic, the status of the crosswalk signal, the creeping advance of the adjacent vehicle. All these regularities can, and are, used to help prepare the motor system for the final act of pressing the pedal. Indeed, research suggests that participants seem able to exploit such environmental regularities in simulated stop-sign detection tasks [22].

Looking-doing latency, while analogous to reaction time in certain ways, may of course involve a number of cognitive abilities not strained by typical reaction time tasks. Latencies to action after a self-initiated move of the view-screen could be improved by anticipating or remembering what is occurring at the location to be fixated (one might propose a similar role for memory in driving performance). Looking-doing latencies may also be improved by better task switching capacities, as a view-screen shift may also reflect a transition, for example, from military to economic considerations. Increases in looking-doing latency with age could be due to declines in a specific ability tapped by looking-doing latency, or due to declines in the capacity to dynamically coordinate these abilities into complex behavior.

Many researchers have attempted to isolate so called domain-general capacities by designing tasks unlike any real world situation, tasks which restrict participants' abilities to initiate and prepare for stimulus presentation. This eliminates means of compensation. Of course, the hope that removing complex environmental contingencies provides any especially deep understanding of human cognition is predicated on the assumption that the exploitation of such contingencies is not pervasive. If exploiting such contingencies is central to virtually all natural cognitive-motor behavior these putative domain-general measures are more likely to be domain-none. Our measure, in contrast, is of direct relevance to the task. Furthermore, looking-doing latency exhibits much more direct relevance to real world tasks, such as food preparation which also seem to be broken down into looking-doing couplets [23], than simple reaction time measures.

There have been some mixed results regarding whether expertise can attenuate specific declines. Some have argued that expertise should attenuate declines most in highly domain-relevant tasks [24], and especially those on measures in which experience shows significant impact on performance. Those researchers have noted that examples of domains where experience does not reduce age-related declines are mostly cases where measures of decline are weakly related to the domain. For example, it is unclear to what extent the speed of check and threat detection on a 4×4 chessboard is relevant to actual chess performance [12]. In the present study we use a measure which is strongly related to expertise. One cannot measure domain performance more directly, and less invasively, than using data that are automatically stored by simply performing, yet we found no evidence that training can attenuate response time declines in a sample of 3305 participants. Instead, our findings are more in keeping with the finding that whether attenuation is possible also depends on the task [25].

While our work does not directly assess the neurobiological bases of age-related decline, the isolation of these changes to the mid-twenties is potentially relevant to this literature. Consider, for example, changes in myelination integrity known to be related to finger tapping speed. These changes are thought to peak around 39 [26], far outside the confidence interval for the declines documented here, and so seem a poor candidate explanation. On the other hand, metabolic changes, such as in ratios of N-acetylaspartate (NAA) to choline (Cho) appear to begin in the early twenties or sooner [27], are thus, logically, more likely candidates.

One of the challenges to isolating the effects of aging is piecing out the age-related declines from experience and skill related effects and also from extraneous factors such as cohort effects. Our results are probably not explained by a cohort effect in the general population (because our sample is probably not representative of that population), or due to a cohort effect regarding video games generally (because the entire sample had access to video games). It is true, however, that cohorts differ respect to how young they were when the first real-time-strategy (RTS) games with a contemporary interface emerged (WarCraft, the ancestor to StarCraft 2, came out in 1994). In other words, it might be possible to explain these with reference to a critical period for RTS skill development.

We take the existence of such a critical period to be rather unlikely because we have strong evidence that the looking-doing latencies of people 30 years of age tend to be slower, yet these individuals would have had access to RTS games at 12 years of age. Any critical period explaining our results would have to be very early in life. However, it will be impossible to empirically test for this period's existence until there are older individuals who did have access to RTS video games at all ages. However, given that there is some concern that our results might be uninteresting if they could be explained away by cohort effects, is fair to note that the existence of critical periods even for highly specialized interfaces would be deeply interesting. It would seem to suggest, for example, that children would need to be provided with experience in whatever computer interfaces they are likely to need as adults. Nevertheless, we take the most likely explanation of our results to be that adults are becoming slower with time.

Importantly, the present methods could be extended to a longitudinal design without confounding skill and training effects [2] as our measures of performance are embedded, and in no way interfere with, the task itself. Such approaches also seem useful for the study of compensation. Our exploratory analysis suggests that telemetric experiments akin to those proposed in Thompson, Blair, Chen & Henrey [1] should compare the costs on older and younger participants by compromising these possible compensatory strategies. Relevant manipulations could include (a) restrictions on the number of available hotkeys (b) the presence of a ‘mini-map’, and (c) the forced use of complex units. StarCraft 2 comes with flexible customization software, that would allow the instantiation of these manipulations in otherwise identical StarCraft 2 games.

Though our sample does not contain adults older than 44, our results suggest that clinical aging research might do well to focus not only on treatments that attenuate declines, but also support the capacity to offload cognitive demands in rich task environments. We found that age was associated with response time declines even when skill is held constant, which suggests that some form of indirect compensation is facilitating the performance of older players. While our compensatory analysis was exploratory, some candidate compensatory mechanisms were observed in the sample.

In summary, we provide the most precise estimate thus far of the onset, around 24 years of age, of cognitive-motor decline in an complex task performed by millions of people around the world. Despite it's early onset, the decline is a significant performance deficit, suggesting early adulthood declines are real world relevant. Further, we find no evidence that this decline can be attenuated by expertise, despite claims that domain relevance should be a major determinant on whether attenuation should occur [24]. Experience nevertheless allows one to compensate for these declines *indirectly*. In our study, older players appear to hold their own despite their declines, perhaps by decreasing their cognitive load through the use of simplified strategies or improved use of the game interface.

At the broadest level, our research, among many others, contributes to a more dynamic portrait of aging. The veneer of stable competence in mid-life masks genuine adult development; cognitive-motor decline begins even in the midst of continuing brain growth [28]. Rather than stability, we have lifelong flux. Our day-to-day performance is, at every age, the result of the constant interplay between change and adaptation.

## Supporting Information

Materials S1 **Supplementary Methods and Materials.**
(DOC)
