# Supplementary material for: Over the Hill at 24: Persistent Age-Related Cognitive-Motor Decline in Reaction Times in an Ecologically Valid Video Game Task Begins in Early Adulthood
Source: PLoS One. 2014 Apr 9;9(4):e94215. doi: 10.1371/journal.pone.0094215 (PMC3981764; doi:10.1371/journal.pone.0094215)
Supplement: Materials S1 — Supplementary Methods and Materials. (DOC) [file pone.0094215.s001.doc]

**Supporting Materials**

*Primary Models*

**Model 1:** Linear regression of log Looking-Doing Latency on League, age, and the League X Age interaction. The response is therefore log milliseconds.

Adjusted R^2^ = 0.4646

RMSE = 0.2044

Obtained Coefficients (non-significant coefficients not reported):

Bronze: 6.76 + 0.008*age

Silver: 6.58 + 0.008*age

Gold: 6.46 + 0.008*age

Platinum: 6.33 + 0.008*age

Diamond: 6.25 + 0.008*age

Masters: 6.00 + 0.008*age

**Model 2:** Piecewise linear regression of log Looking-Doing Latency including coefficients for League, Age, and for YearsOver24 (years of age over model 2’s best estimate of k, 24). The response is therefore log milliseconds.

Adjusted R^2=^=0.4663

RMSE=0.2041

Obtained Coefficients (non-significant coefficients not reported):

Bronze: 6.87 + 0.01* YearsOver24

Silver: 6.69 + 0.01* YearsOver24

Gold: 6.56 + 0.01* YearsOver24

Platinum: 6.42 + 0.01* YearsOver24

Diamond: 6.32 + 0.01* YearsOver24

Masters: 6.03 + 0.01* YearsOver24

*Exploratory Models (models 3-12)*

*Note: Intercepts varied by league in all of the following models.*

**Model 3:** Total Hours: Non-significant

**Model 4:** Weekly Hours: -0.44 +/- 0.08 hours per week. R^2 = 0.078. RMSE = 11.26

**Model 5:** Unique Hotkeys: +0.033 +/- 0.018 unique hotkeys in a game. R^2 = 0.106. RMSE = 2.198

**Model 6:** Complex Units per Second: -0.0001 +/- 0.00008 complex units per second. R^2 = 0.03. RMSE = 0.001.

**Model 7:** Complex Ability per Second: -0.00032 +/- 0.000002 complex abilities per second. R^2 = 0.025. RMSE = 0.009.

**Model 8:** Distance between Looking-Doing-Cycles: + 0.002 +/- 0.0001 seconds. R^2 = 0.2897. RMSE = 0.1622.

**Model 9:** RC Offscreen: Non-significant

**Model 10:** Hotkey Selects per Second -0.0073 +/- 0.0030 hotkey selects. R^2 = 0.1971, RMSE = 0.372

**Model 11:** Hotkey Assigns per Second: -0.00018 +/- 0.00013 hotkey assigns. R^2 = 0.229, RMSE = 0.016

**Model 12:** Offscreen attacks per Second: 0.00027 +/- 0.000010 offscreen attacks. R^2 = 0.072, RMSE = 0.013

*Term Definitions*

*Note that these definitions are adapted from Thompson, Blair, Chen, & Henrey (2013). For a complete overview of the replay parsing procedure, see Thompson, Blair, Chen, & Henrey (2013).*

League

The leagues "Bronze", "Silver", "Gold", "Platinum", "Diamond", and "Masters”, coded 1:6

Off-screen

Commands to game coordinates that were either (a) farther east or west than 20 game coordinates from screen centre, (b) farther north than 15.5 game coordinates from screen centre, or (c) farther south than 6 coordinates from screen centre. Commands at off-screen locations must be issue by clicking on the mini-map.

Looking-Doing-Cycle

Screen-movement data was aggregated into screen fixations using a fixation-IDT algorithm[19]. The dispersion threshold was set to 6 game coordinates and the duration threshold to 20 timestamps (roughly 230 milliseconds). Looking-doing-cycles are screen-fixations with at least one action.

*Variable Definitions*

*Note that these definitions are adapted from Thompson, Blair, Chen, & Henrey (2013). For a complete overview of the replay parsing procedure, see Thompson, Blair, Chen, & Henrey (2013).*

Looking-Doing-Latency

Mean Latency (in milliseconds) to first action within a Looking-Doing-Cycles.

Distance between Looking-Doing-Cycles

Mean distance (in milliseconds) between Looking-Doing-Cycles.

Total Hours

Participant reports of how many hours they have played StarCraft 2.

Hours Per Week

The product of participant reports regarding how many hours they typically play StarCraft 2 per day and how many days they typically play per week.

*Hotkey Variables*

Assign to Hotkeys per second

Players may assign a collection of units to a key from 0 to 9 at any time. The value of this variable refers to number of assignments per second.

Select by Hotkeys per second

Once a collection of units has been assigned to a hotkey, players may select those units at any time in order to give them orders. The value of this variable refers to the number of these selections per second.

Unique Hotkeys

Unique hotkeys refers to the number of keys used for assignment throughout the game.

Complex Unit Production and Use Variables

Complex Ability Use per second:

The number of ground or unit targeted abilities per game second (abilities listed as C in our ability coding). These abilities require the player to click, with their mouse, on a specific location or game unit. This is also typically done under time constraints.

Complex Units Made per second

Number of "ghosts", "infestors", and "high templar" units trained by the player per second.

Unique Units Made

Number of different unit types (e.g. ‘Probes’, ‘Marines’, ‘Roaches’) produced in game.

Workers Made per second

The count of workers (a "Probe", "Drone", or "SCV") created by a player per second. This units production is required yet is relatively independent from the other activities of the player. Its production can therefore be thought of as a dual-task scenario that all players must overcome.

*Direct measures of Attentional Control*

Minimap Attacks per second

Number of attack commands that were "off-screen" per second. This behavior requires attention to a small version of the overall game map in the lower corner of the screen. It provides important general information about the current game state.

Minimap right-clicks

Number of right-click ("contextual-key") commands that were "offscreen" (see definition of terms) per second. This behavior requires attention to a small version of the overall game map. It provides important general information about the current game state.

Abilities Coding

*Note that these definitions are adapted from Thompson, Blair, Chen, & Henrey (2013). For a complete overview of the replay parsing procedure, see Thompson, Blair, Chen, & Henrey (2013).*

This is a characterization of the special abilities outputted by the replay parsing software, SC2Gears. We code them as complex or not complex (Complex=C; Not Complex=N). The list is also included to allow for reproduction of our analysis.

250mm Strike Cannons (Thor) C

Activate/Deactivate Auto-attack Structur N

Activate/Deactivate Auto-repair (MULE) N

Activate/Deactivate Auto-repair (SCV) N

Archon Warp (High Templar or Dark Templa N

Arm Silo with Nuke (Ghost Academy) N

Assault Mode (Viking) N

Attack N

Attack (Bunker) N

Blink (Stalker) C

Burrow (Baneling) N

Burrow (Drone) N

Burrow (Hydralisk) N

Burrow (Infested Terran) N

Burrow (Infestor) N

Burrow (Queen) N

Burrow (Roach) N

Burrow (Ultralisk) N

Burrow (Zergling) N

Calldown Extra Supplies (Orbital Command N

Calldown MULE (Orbital Command) N

Cancel N

Cancel Brood Lord Morphing (Corruptor) N

Cancel Graviton Beam C

Cancel Greater Spire Upgrade (Spire) N

Cancel Hive Upgrade (Lair) N

Cancel Lair Upgrade (Hatchery) N

Cancel Orbital Command Upgrade (Command N

Cancel Overseer Morphing (Overlord) N

Cancel Planetary Fortress Upgrade (Comma N

Cancel Tac Nuclear Strike (Ghost) N

Cancel an Addon (Barracks) N

Cancel an Addon (Factory) N

Cancel an Addon (Starport) N

Chrono Boost (Nexus) N

Cancel Baneling Morphing (Zergling) N

Cloak (Banshee) N

Cloak (Ghost) N

Contaminate (Overseer) C

Corruption (Corruptor) C

Decloak (Banshee) N

Decloak (Ghost) N

EMP Round (Ghost) C

Explode (Baneling) N

Feedback (High Templar) C

Fighter Mode (Viking) N

Force Field (Sentry) C

Fungal Growth (Infestor) C

Gather Resources Protoss (Probe) N

Gather Resources Terran (SCV) N

Generate Creep (Overlord) N

Graviton Beam (Phoenix) C

Guardian Shield (Sentry) N

Halt (on building) N

Halt (on unit) N

Heal (Medivac) N

Hold fire N

Hold position N

Infested Terran (Infestor) C

Land (Barracks) N

Land (Command Center) N

Land (Factory) N

Land (Orbital Command) N

Land (Starport) N

Leave game N

Lift Off (Barracks) N

Lift Off (Command Center) N

Lift Off (Orbital Command) N

Lift Off (Starport) N

Load (Bunker) N

Load (Warp Prism) N

Load All(Command Center/Planetary Fortre N

Lower Supply Depot (Supply Depot) N

Mass Recall (Mothership) C

Morph to Baneling (Zergling) N

Morph to Brood Lord (Corruptor) N

Morph to Overseer (Overlord) N

Move N

Mutate into Greater Spire (Spire) N

Mutate into a Gateway (Warp Gate) N

Neural Parasite (Infestor) C

Patrol N

Phasing Mode (Warp Prism) N

Psionic Storm (High Templar) C

Raiser Supply Depot (Supply Depot) N

Repair (MULE) N

Repair (SCV) N

Research Protoss Blink N

Research Protoss Charge N

Research Protoss Extended Thermal Lance N

Research Protoss Gravitic Booster N

Research Protoss Gravitic Drive N

Research Protoss Graviton Catapult N

Research Protoss Psionic Storm N

Research Protoss Warp Gate N

Research Terran 250mm Strike Cannons N

Research Terran Behemoth Reactor N

Research Terran Caduceus Reactor N

Research Terran Cloaking Field N

Research Terran Combat Shield N

Research Terran Concussive Shells N

Research Terran Corvid Reactor N

Research Terran Durable Materials N

Research Terran Infernal Pre-Igniter N

Research Terran Moebius Reactor N

Research Terran Nitro Packs N

Research Terran Personal Cloaking N

Research Terran Seeker Missile N

Research Terran Siege Tech N

Research Terran Stimpack N

Research Terran Weapon Refit N

Research Zerg Adrenal Glands N

Research Zerg Burrow N

Research Zerg Centrifugal Hooks N

Research Zerg Chitinous Plating N

Research Zerg Glial Reconstitution N

Research Zerg Grooved Snipes N

Research Zerg Metalic Boost N

Research Zerg Neural Parasite N

Research Zerg Pathogen Glands N

Research Zerg Pneumatized Carapace N

Research Zerg Tunneling Claws N

Research Zerg Ventral Sacks N

Return Cargo (Drone) N

Return Cargo (MULE) N

Return Cargo (Probe) N

Return Cargo (SCV) N

Root (Spine Crawler) N

Root (Spore Crawler) N

Salvage (Bunker) N

Scan Move N

Scanner Sweep (Orbital Command) N

Seeker Missile (Raven) C

Set Alliance N

Set Rally Point (Command Center) N

Set Rally Point (Factory) N

Set Rally Point (Hatchery) N

Set Rally Point (Nexus) N

Set Worker Rally Point (Hatchery) N

Set rally point N

Siege Mode (Siege Tank) N

Sniper Round (Ghost) C

Spawn Changeling (Overseer) C

Spawn Creep Tumor (Queen) N

Spawn Larva (Queen) N

Stimpack (Bunker) N

Stimpack (Marauder) N

Stimpack (Mixed Units) N

Stop N

Stop (Bunker) N

Stop Generating Creep (Overlord) N

Tac Nuclear Strike (Ghost) C

Tank Mode (Siege Tank) N

Transfusion (Queen) C

Transport Mode (Warp Prism) N

Unburrow (Baneling) N

Unburrow (Drone) N

Unburrow (Hydralisk) N

Unburrow (Infestor) N

Unburrow (Queen) N

Unburrow (Roach) N

Unburrow (Ultralisk) N

Unburrow (Zergling) N

Unload All (Bunker) N

Unload All (Command Center/Planetary For N

Unload All (Nydus Worm) N

Unload All (Warp Prism) N

Unload All At (Medivac) N

Unload All At (Overlord) N

Unload All At (Warp Prism) N

Unload All (Medivac) N

Unload All (Overlord) N

Unload All (Warp Prism) N

Unload Unit (Bunker) N

Unload Unit (Command Center/Planetary Fo N

Unload Unit (Medivac) N

Unload Unit (Nydus Worm) N

Unload Unit (Overlord) N

Unload Unit (Warp Prism) N

Upgrade Protoss Air Armor 1 N

Upgrade Protoss Air Armor 2 N

Upgrade Protoss Air Armor 3 N

Upgrade Protoss Air Weapons 1 N

Upgrade Protoss Air Weapons 2 N

Upgrade Protoss Air Weapons 3 N

Upgrade Protoss Ground Armor 1 N

Upgrade Protoss Ground Armor 2 N

Upgrade Protoss Ground Armor 3 N

Upgrade Protoss Ground Weapons 1 N

Upgrade Protoss Ground Weapons 2 N

Upgrade Protoss Ground Weapons 3 N

Upgrade Protoss Shield 1 N

Upgrade Protoss Shield 2 N

Upgrade Protoss Shield 3 N

Upgrade Terran Building Armor N

Upgrade Terran Hi-sec Auto Tracking N

Upgrade Terran Infantry Armor 1 N

Upgrade Terran Infantry Armor 2 N

Upgrade Terran Infantry Armor 3 N

Upgrade Terran Infantry Weapons 1 N

Upgrade Terran Infantry Weapons 2 N

Upgrade Terran Infantry Weapons 3 N

Upgrade Terran Neosteel Frame N

Upgrade Terran Ship Plating 1 N

Upgrade Terran Ship Plating 2 N

Upgrade Terran Ship Plating 3 N

Upgrade Terran Ship Weapons 1 N

Upgrade Terran Ship Weapons 2 N

Upgrade Terran Ship Weapons 3 N

Upgrade Terran Vehicle Plating 1 N

Upgrade Terran Vehicle Plating 2 N

Upgrade Terran Vehicle Plating 3 N

Upgrade Terran Vehicle Weapons 1 N

Upgrade Terran Vehicle Weapons 2 N

Upgrade Terran Vehicle Weapons 3 N

Upgrade Zerg Flyer Attacks 1 N

Upgrade Zerg Flyer Attacks 2 N

Upgrade Zerg Flyer Attacks 3 N

Upgrade Zerg Flyer Carapace 1 N

Upgrade Zerg Flyer Carapace 2 N

Upgrade Zerg Flyer Carapace 3 N

Upgrade Zerg Ground Carapace 1 N

Upgrade Zerg Ground Carapace 2 N

Upgrade Zerg Ground Carapace 3 N

Upgrade Zerg Melee Attacks 1 N

Upgrade Zerg Melee Attacks 2 N

Upgrade Zerg Melee Attacks 3 N

Upgrade Zerg Missile Attacks 1 N

Upgrade Zerg Missile Attacks 2 N

Upgrade Zerg Missile Attacks 3 N

Upgrade to Orbital Command (Command Cent N

Upgrade to Planetary Fortress (Command C N

Upgrade to Warp Gate (Gateway) N

Uproot (Spine Crawler) N

Uproot (Spore Crawler) N

Use ability 5f01 N

Use ability 6e00 N

Use ability 7b42 N

Use ability 12940 N

Use ability 14442 N

Use ability 15d09 N

Use ability 17200 N

Use ability 17801 N

Use ability 17901 N

Use ability 1fa0e N

Use ability 30422 N

Vortex (Mothership) C

Yamato Cannon (Battlecruiser) C

Tab N
